# Radiomic analysis of contrast-enhanced ultrasound data

**DOI:** 10.1038/s41598-018-29653-7

**Published:** 2018-07-27

**Authors:** Benjamin Theek, Tatjana Opacic, Zuzanna Magnuska, Twan Lammers, Fabian Kiessling

**Affiliations:** 1 Institute for Experimental Molecular Imaging, Center for Biohybrid Medical Systems, RWTH Aachen University Clinic, Forckenbeckstraße 55, 52074 Aachen, Germany; 2 Comprehensive Diagnostic Center Aachen, RWTH Aachen University Clinic, Pauwelsstr. 30, 52074 Aachen, Germany

## Abstract

Radiomics describes the use radiological data in a quantitative manner to establish correlations in between imaging biomarkers and clinical outcomes to improve disease diagnosis, treatment monitoring and prediction of therapy responses. In this study, we evaluated whether a radiomic analysis on contrast-enhanced ultrasound (CEUS) data allows to automatically differentiate three xenograft mouse tumour models. Next to conventional imaging biomarker classes, i.e. intensity-based, textural, and wavelet-based features, we included biomarkers describing morphological and functional characteristics of the tumour vasculature. In total, 235 imaging biomarkers were extracted and evaluated. Dedicated feature selection allowed us to identify user-independent and stable imaging biomarkers for each imaging biomarker class. The selected radiomic signature, composed of *median image intensity, energy* of *grey-level co-occurrence matrix, vessel network length*, and *run length nonuniformity of the grey-level run length matrix from the diagonal details*, was used to train a linear support vector machine (SVM) to classify tumour phenotypes. The model was trained by using a four-fold cross-validation scheme and achieved 82.1% (95% CI [0.64 0.92]) correct classifications. In conclusion, our results show that a radiomic analysis can be successfully performed on CEUS data and may help to render ultrasound-based tumour imaging more accurate, reproducible and reliable.

## Introduction

The huge geno- and phenotypic variability of tumours poses the question if better patient stratification and precision medicine can help to improve the therapeutic outcome^1–4^. For molecularly targeted therapy approaches, diagnostic test kits have been shown to identify patients, which are more likely to benefit from the respective therapy^5^. The Biotechnology Innovation Organization identified in their meta-analysis about the success rate of clinical trials a three-fold higher likelihood of a Phase I to approval transition when tissue biomarkers were used as inclusion or exclusion criteria for patient enrolment^6^. However, for many therapies tissue biomarkers for patient stratification are not available. In this context, biomarkers may also derive from non-invasive imaging data and describe morphological, pathophysiological and molecular characteristics of tumours^7–10^.

Advanced medical image analysis algorithms, especially for pattern recognition, allow the extraction of quantifiable imaging biomarkers from clinical routine imaging data. The emerging field of radiomics makes use of this development by mining quantifiable features based on tumour image intensity, shape, and texture to characterize the tumour phenotype and relate it to a clinical outcome^11–15^. Once these correlations are established, it is thought that they can be used for precision medicine and patient pre-selection. So far, mainly four imaging biomarker classes, i.e. tumour intensity, tumour morphology, tumour texture and wavelet-based features, were extracted from non-contrast enhanced CT and MRI images and used in radiomics analyses^12,13^. These imaging techniques have beneficial properties such as low user dependency, high reproducibility, and are tomographic, covering the complete tumour. Most recent radiomic studies have been trying to complement anatomical with functional information gained from contrast-enhanced scans^16–19^. This functional information enables a more comprehensive description of the pathophysiology of tumours and thus may improve the diagnostic power of radiomics analysis, e.g. for selecting the most appropriate therapy.

Similar to the post-processing of images from other medical imaging devices also ultrasound (US) image post-processing has a long history. However, first order statistics, texture analysis and especially morphological parameters of the tumour vasculature were mostly handled separately, even though a single contrast-enhanced US (CEUS) scan suffices to extract all these information at once. Each of these imaging biomarker classes has proven its value for disease diagnosis, staging or prediction and evaluation of therapy responses during anti-tumour therapy^14,20–25^. For example, it was shown that the microvascular structure, as determined by CEUS, could be used to differentiate benign and malignant breast lesions^20^. In another study, Lowerison and colleagues developed a statistical model to analyse first-order speckle statistics from CEUS data of tumours^22^. They were able to infer functional and structural information from these scans to assess anti-angiogenic therapy responses. Similarly, textural features extracted from the margins and cores of breast tumours showed a high predictive accuracy for the response to neoadjuvant chemotherapy and survival^23^. Guo *et al*. combined intensity-based, shape-based, textural- and wavelet-based parameters to evaluate the correlation of quantitative US features and biological characteristics^26^. The combination of parameters from six BI-RADS categories generated the best classification model, which was trained to discriminate hormone receptor-positive and HER2-negative tumours. Thus, combining parameters from these imaging classes can have a synergistic effect and aid diagnosis.

In order to test the suitability of using CEUS images for radiomics analysis, we extracted quantitative imaging features from first-order statistics, textural analysis, morphological and functional characterization of the tumour vasculature, and the wavelet-transform. It is the first study systematically integrating morphological and functional information of the tumour vasculature in a radiomic analysis. We identified the most stable parameters, having the highest discriminative power, for each of these four imaging biomarker classes, and used them to develop an automated tumour classification model. Subsequently, the tumour classification model was tested for its ability to discriminate three different tumour xenograft mouse models.

## Results

The measurements were performed with a preclinical high-frequency US system (Vevo2100, FUJIFILM VisualSoncis, Toronto, Canada). First order statistics, textural parameters, as well as wavelet-based features, were calculated on the basis of B-mode images, which underwent a wavelet-based filtering technique for noise removal. Time-intensity curves from CEUS scans were used to calculate functional parameters of the vasculature. Every mouse was imaged four times (two times at two different positions). Three users independently delineated the tumours in all scans.

### Image analysis

**Figure 1:**
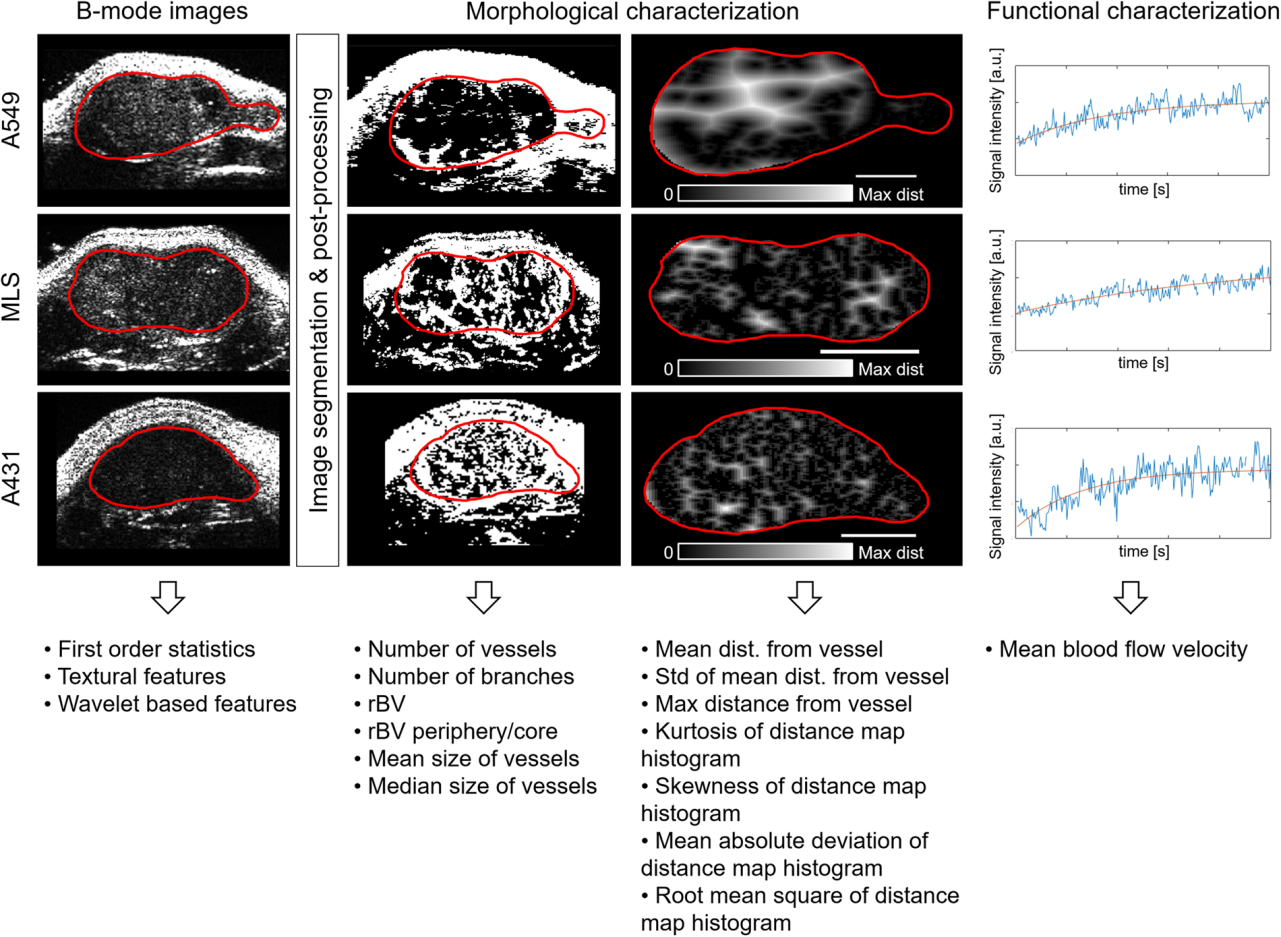
Image biomarker extraction from A549, MLS and A431 tumours. B-mode images were used to calculate first order statistics, textural features and wavelet-based features of the tumours. Morphological features characterizing the tumour vasculature were extracted from an automated blood vessel segmentation algorithm and its corresponding distance map. The scale bars correspond to 1 mm. Functional characteristics of the tumour vasculature were obtained by using the time intensity curves of the acquired contrast-enhanced ultrasound data.

For each individual CEUS scan, a total of 235 imaging features were extracted. The calculation of the first order statistics (14 features), textural features (30 features), parameters of the tumour vasculature (15 features) and wavelet-based features (176 features) was performed automatically after the tumour boundaries were manually delineated by a user. First order statistics, textural features and wavelet-based features were obtained from I) B-mode images. The morphological and functional characterization of the tumour vasculature was based on II) vessel segmentations, III) distance maps, which were calculated on the basis of the binary vessel segmentation and displaying the distance of each pixel to the nearest vessel, and IV) destruction replenishment time intensity curves to calculate the blood flow velocity (Fig. 1).

### Unsupervised classification

**Figure 2:**
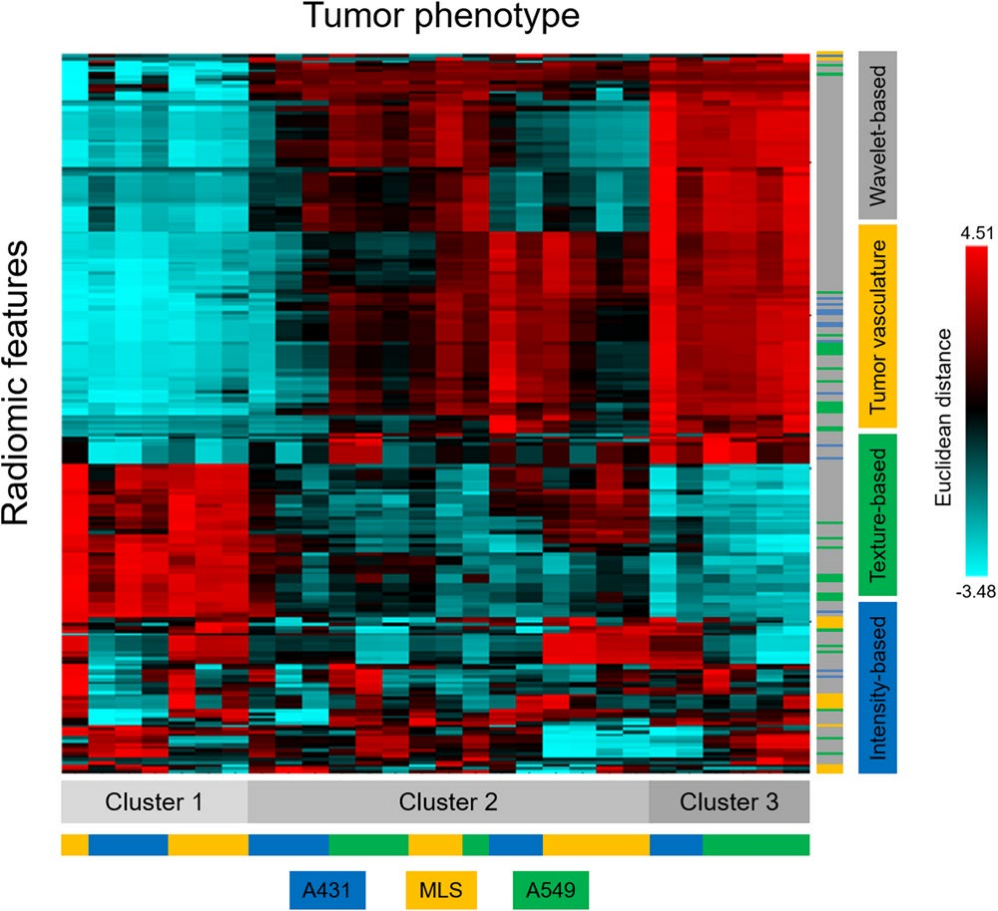
Unsupervised tumour classification. Cluster image map of all 235 radiomics features and 28 US measurements. The overall arrangement is based on the Euclidean distance (color coding) and average linkage theorem. Based on all radiomics features, three general clusters can be identified, as depicted at the bottom. The colour bars are representing the tumour model (bottom) or image biomarker class (right).

Dataset 1, consisting of 235 features for each of the 28 US scans, was used for an unsupervised clustering approach. The clustering of radiomic features enabled the identification of three clusters with similar radiomic expression pattern (Fig. 2). No strict discrimination of the three tumour models was possible. However, the likelihood of the tumour models to be part of a certain cluster was not randomly distributed. The multinomial test revealed a statistically significant underrepresentation of A431 (p = 0.01) and MLS tumours (p = 0.0001) in cluster 3, as well as A549 tumours (p = 0.0005) in cluster 1.

### Feature selection

**Figure 3:**
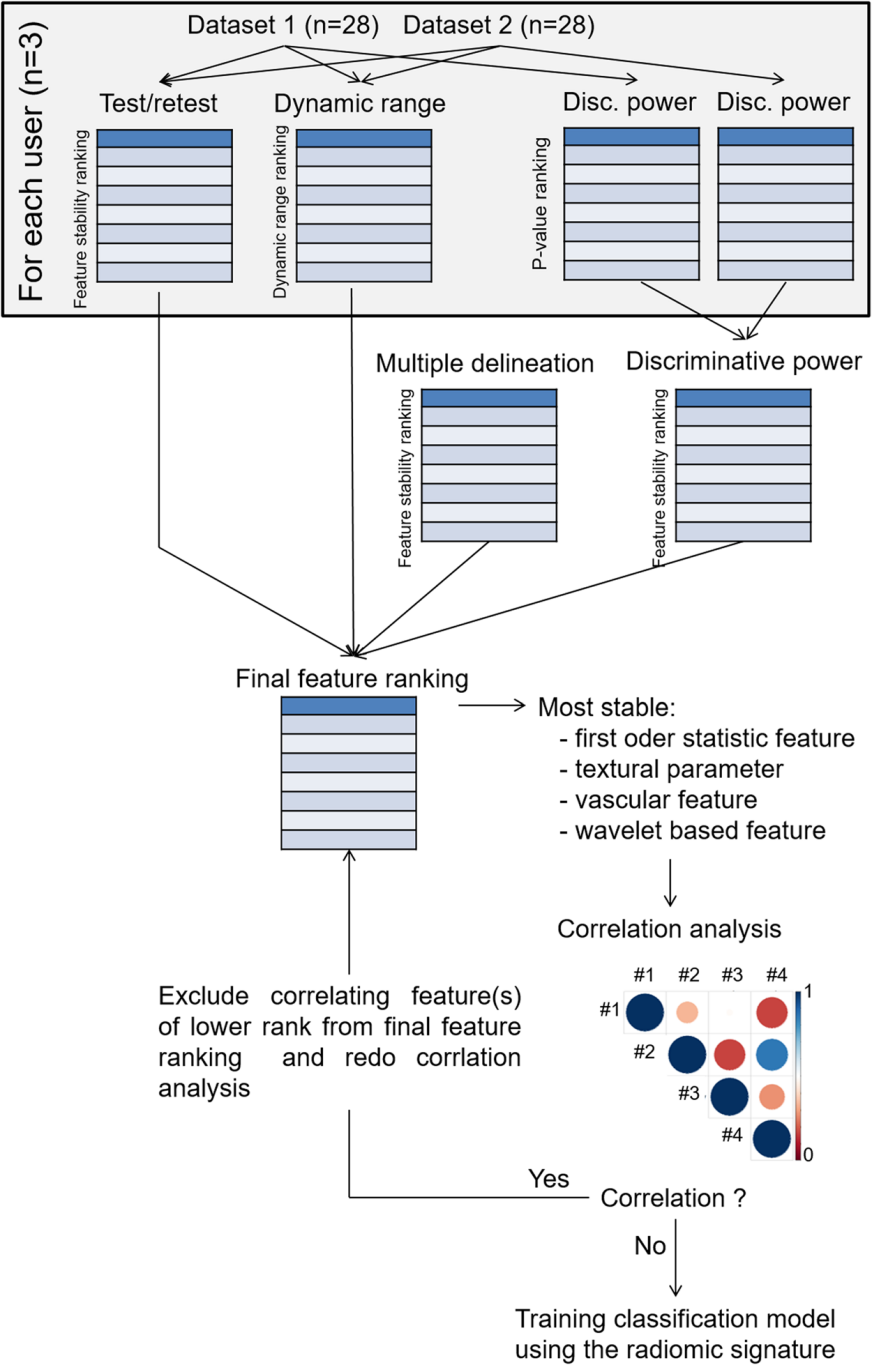
Workflow of feature selection process. Two datasets were used to determine the feature stability by performing a Test/Retest measurement, and ranking all extracted features with respect to their stability in between the two measurements. The dynamic range in relation to the variability of the measurement was assessed and ranked from high to low. To identify features, which are user independent, the two datasets of each user were combined, and tested for their stability in between the different users. Furthermore, the Kruskal-Wallis test was performed on each dataset to generate a discriminative power ranking. The Test/Retest, dynamic range, multiple delineation and discriminative power rankings were applied to compute a final ranking of all extracted imaging features. The most stable features of each imaging biomarker class were selected to compose the radiomic signature. A correlation analysis was performed to exclude correlating features. The final radiomic signature consisted of four features which were not correlating, one from each imaging biomarker class.

**Figure 4:**
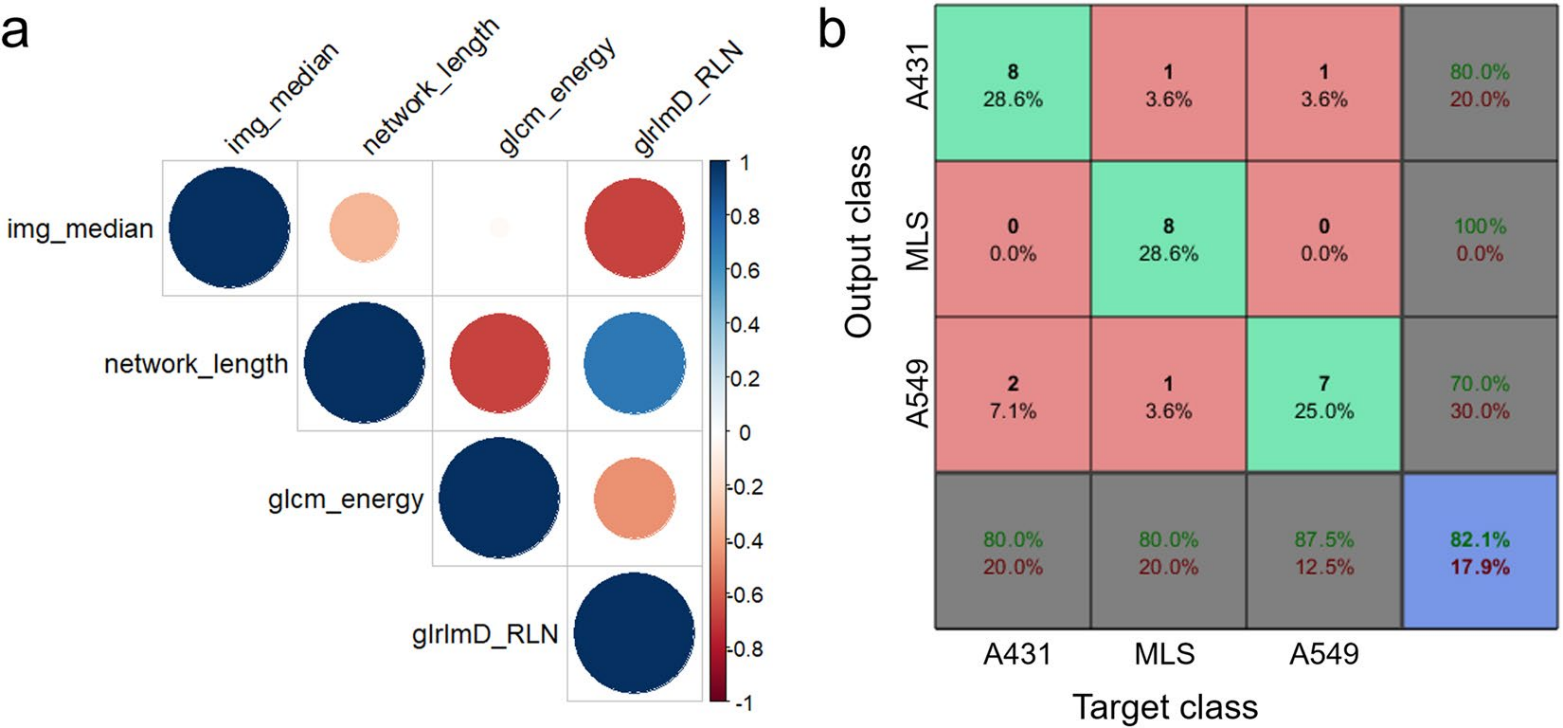
Cross-correlation of radiomic signature and supervised tumour classification. The Pearson correlation coefficient was calculated for each of the imaging biomarker pairs. If a feature pair had a value of higher than 0.75 it was excluded and replaced by the next highest ranked feature of the same imaging biomarker class. The correlation of the final radiomics signature is depicted in (**a**). The confusion matrix is based on the training of a linear support vector machine using a 4-fold cross-validation scheme (**b**). 82.1% of the classified mice were assigned to the correct output class.

To identify a set of features (radiomics signature), which can be used to automatically differentiate the three tumour models, a dedicated feature selection process was developed and applied to the data (Fig. 3). The feature stability in separate measurements at the same location was evaluated using a Test/Retest analysis, calculating the concordance correlation coefficient (CCC). All features were ranked in accordance with their CCC, from high to low. The impact of the tumour delineation was assessed by comparing the feature similarity of the segmentations performed by the three different users. A high feature similarity between the users is a measure for the user-independence of the feature. A ranking from high to low was prepared as well. As a measure of the biological variability in relation to the measurement variability, we calculated the normalized dynamic range of the extracted parameters, and ranked the features from high to low. A fourth ranking, which was based on the discriminative power of the individual features, was calculated as well. This was done by the Kruskal-Wallis test, and features were ranked from low to high p-value. The four rankings, describing feature stability, dynamic range, user-independence, and discriminative power were the basis for a final feature ranking. The highest ranked features for each imaging biomarker class were: I) image median, II) vessel network length, III) run length nonuniformity of the grey-level run length matrix (glrlm_RLN), and IV) the run length nonuniformity of the diagonal grey-level co-occurrence matrix (glcmD_RLN). A correlation analysis revealed a strong correlation of the features from biomarker class III and IV (r-value > 0.75). As the features of the final radiomic signature should not correlate, the feature with the lower rank, i.e. glrlm_RLN was replaced by the feature with the next highest rank from the same imaging biomarker class until the r-value between the parameters dropped below 0.75. The final radiomics signature was I) image median, II) vessel network length, III) energy of the grey-level co-occurrence matrix (glcm_energy), and IV) the run length nonuniformity of the diagonal grey-level co-occurrence matrix (glcmD_RLN; Fig. 4a).

### Automated tumour classification

A linear support vector machine (SVM) classification model was selected for tumour classification using the above mentioned radiomic signature as input parameters. To assess the classification performance, we used Dataset 1 (n = 28) and performed a 4-fold cross-validation. The model obtained a validation-accuracy of 82.1% (95% CI [0.64 0.92]; Fig. 4b), giving us good confidence that the trained model is able to classify the three different tumour phenotypes.

## Discussion

In this study we were able to show that CEUS scans can be used to perform a radiomics analysis, enabling the classification of three different tumour xenograft models. Therefore, we did not only rely on the imaging biomarker classes tumour intensity, tumour texture, and wavelet-based features, but included morphological and functional parameters of the tumour vasculature as well. We expected a synergistic effect of adding vascular parameters to our analysis, because several studies have already shown the importance of the tumour vasculature with respect to tumour lesion classification and staging, the detection of therapy responses, and the tumour accumulation of drugs^7,20,21,27^. Furthermore, also other radiomic studies started to include contrast-enhanced scans in their analysis, to cover the homo- and heterogeneity of functional aspects, such as glucose metabolism (18^F^-FDG PET), perfusion (CT perfusion) or blood vessel permeability (DCE-MRI), of the tumour^16–19^. The combination of anatomical and functional information might enable the development of better models for computer-aided diagnosis.

The rational of our feature selection process was to identify parameters that are considerably stable in repetitive measurements and between different observers. Thus, data mining was performed to generate a feature ranking which allowed us to identify a radiomic signature with high discriminative power and high robustness. From each imaging biomarker class one feature was selected to train a linear SVM classification model. The selected first order statistics feature, the median signal intensity of the image, is a measure of the scattering structures present in the tumour. The textural feature, energy of the GLCM, can be considered as a parameter of homogeneity, as higher values are associated with a more constant image. The vascular network length is simply describing the overall length of the vascular network. The wavelet-based feature, run length nonuniformity of the diagonal grey-level run length matrix, is a measure of the signal intensities at which most of the runs occur. The higher the value, the more runs at higher intensities were measured. Taking the nature of intensity-based and textural parameters into consideration, it is of high importance that the measurements are standardized and reproducible, to avoid systematic errors in the analysis.

Some limitations with respect to the current study setup exist: (1) We were only imaging arbitrary slices of subcutaneous tumours and did not expect heterogeneous tumour shapes. Therefore, shape-based criteria were excluded from our analysis. In a clinical scenario, shape-based features should be included in the analysis as well. (2) Preclinical tumour models are less heterogeneous in comparison to the clinical situation. Identifying suitable clinical datasets, which enable a retrospective analysis, would be the next step to show that also clinical CEUS data are suitable for radiomic analysis approaches. (3) Only a relatively low number of animals was tested, which nevertheless gave already good confidence that CEUS data can be used to perform a radiomic analysis. Future studies should solve the above mentioned issues and could be improved even more by using matrix transducers, which would enable the acquisition of 3D CEUS data. This would also allow a more reliable assessment of the tumour morphology, and enable the inclusion of relevant morphological biomarkers as well.

It has to be kept in mind, that in this study a standardized acquisition protocol was used to image all mice. This includes the settings of the ultrasound machine, positioning of the transducer, injection of contrast agent and image acquisition. In the clinical setting such a strict standard operating procedure is more difficult to implement, because several factors, e.g. patient habitus and movement, can only partially be controlled. However, such deviations might have a strong influence on the extracted features. Consequently, the feature selection process will most likely present different radiomic signatures for different types of examinations. In case the workflow is kept constant, and is in line with a predefined standard operating procedure, the likelihood of gaining relevant information by a radiomic analysis is highest.

Overall, this study shows that CEUS imaging can be used for computer-aided diagnosis. In this context, by including morphological and functional vascular parameters of the tumours in a radiomics approach, automated discrimination of the different tumour xenografts was achieved with an accuracy of 82.1%. In future studies, we will investigate whether such considerably high diagnostic accuracies can also be obtained from clinical CEUS data, e.g. for tumour characterization and the assessment of therapy responses.

## Materials and Methods

All animal experiments performed in this manuscript were conducted in accordance with the animal welfare guidelines and approved by the State Agency for Nature, Environment and Consumer Protection NRW (LANUV). Dataset 1 comprises 9 CEUS scans which were additionally used in another publication, detailing the vessel segmentation algorithm^28^. Three users (B.T., T.O., and F.K.) independently segmented the tumours, to analyse the feature stability among different segmentations.

### Microbubble Synthesis

The poly(butyl cyanoacrylate) (PBCA) microbubble (MB) synthesis and characterization were performed as described in^29^. Coulter counter measurements revealed a mean MB diameter of ~2 µm.

### Contrast-enhanced ultrasound imaging

Tumor-bearing mice were anesthetized during the whole imaging process, using 2% (v/v) isoflurane. Two transversal slices of the tumour were imaged, using the Vevo 2100 imaging system (FUJIFILM Visualsonics, Toronto, Canada) in combination with the MS550 Transducer (FUJIFILM Visualsonics, Toronto, Canada), operating at 40 MHz. After the i.v. injection of 1*10^7^ MB a destruction-replenishment sequence was acquired. The sequence acquires B-mode images with 2% power, resulting in non-destructive imaging, before and after a destructive pulse, which eliminates the MB in the imaging plane.

### Post Processing and Image Analysis

**Figure 5:**
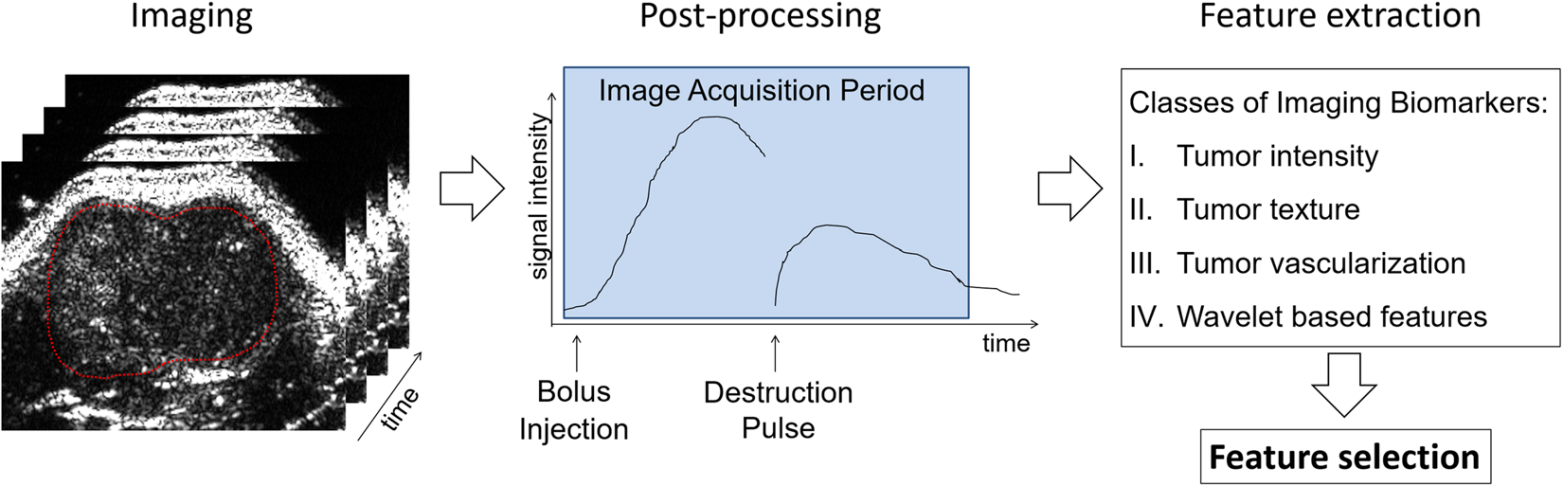
Schematic presentation of the imaging and analysis procedure. Two transversal planes of each tumour were recorded over time. Shortly after the image acquisition was started, microbubbles were injected as a bolus. A high mechanical index destruction pulse was applied and the replenishment of microbubbles was recorded. The raw RF B-mode data was post-processed to enable feature extraction. All extracted features were then fed into the feature selection process to generate a radiomics signature, which can be used for classification.

Linear B-mode images were directly computed from the radiofrequency (RF) data acquired by the US system, using an algorithm from FUJIFILM VisualSonics. Further post-processing and feature extraction, as schematically depicted in Fig. 5, was performed using Matlab 2017b and R.

### Noise removal

A wavelet transformation was used to remove speckle noise. In brief, B-mode images were log-transformed and the wavelet decomposition at level three was computed, using a biorthogonal wavelet (i.e. bio3.7). The Bayes threshold was calculated and soft thresholding was performed on the detail coefficients of the third level of decomposition. The thresholded detail coefficients were used for the following wavelet reconstruction. After inverting the log-transform, the filtered B-mode images were used for further analysis.

### Vessel segmentation

The segmentation of tumour blood vessels was a prerequisite for the characterization of the tumour vasculature. The semi-automated segmentation algorithm used in this study is described in more detail in^28^. In brief: At first, a manual delineation of the region-of-interest (ROI) was required as user input. All following steps did not need any user input and ran automatically. An adaptive filtering (wiener2) with a kernel size of 3 × 3 pixels was applied to smoothen the image. Subsequent motion tracking by frame differencing enabled the identification of moving microbubbles in the tumour. Furthermore, this process allowed the detection of breathing artefacts, and the respective frames were excluded during the following steps of analysis. The dataset was then subjected to a maximum intensity over time projection, to obtain a 2D grayscale images of the tumours. Noise removal by adaptive thresholding, binarization, and removal of speckle were the final steps of the algorithm to segment the vasculature of the tumours. The vessel segmentation was the input for the morphological characterization of the tumour vasculature.

### Feature extraction

In this study first-order statistics, textural features, anatomical and functional characteristics of the tumour vasculature and wavelet-based features were extracted from each individual dataset, resulting in a total of 235 quantifiable imaging features. The calculation of first order statistics and texture analysis was performed on the manually delineated ROI. Textural features were extracted in accordance to literature^30–33^. The morphological features were based on the binary segmentation of the tumour vasculature. The features analysed in this study are the number of vessels, which were the number of unconnected objects detected in the delineated ROI. The mean and median vessel size describes the mean and median number of pixels of all identified objects, respectively. The vascular network length was the number of positive pixels after skeletonizing the binary vessel segmentation. Based on the vessel segmentation a distance map was calculated to determine the mean distance of pixels, which were not identified as vessels, from the nearest vessel (mean distance from vessel). Additionally, the standard deviation of the mean distance from vessel and the maximum distance from the nearest vessel were obtained from the distance map. The relative blood volume (rBV) calculation of the tumour was based on the number of pixels segmented as vessel, divided by the number of pixels from the whole ROI. The rBV of the outer 50% of the tumour divided by the rBV of the inner 50% of the tumour (rBV periphery/core) was calculated in accordance to the before mentioned formula. The destruction replenishment sequence allowed the calculation of the mean blood flow velocity in the tumour, similar to^34^. Undecimated wavelet-transform, using the coiflet 1 wavelet, was performed on the original B-mode US images. For each individual decomposition first order statistics and textural parameters were calculated as described above.

### Data analysis

Two datasets were available to train, validate and test the classification model. Dataset 1 consisted of 28 CEUS scans, which were recorded in 14 different mice bearing subcutaneous tumours (A431, n = 5; MLS, n = 5; A549, n = 4). Dataset 2 contained 28 CEUS scans of the same tumours at the same positions as in Dataset 1 that were acquired in a second measurement.

### Unsupervised Classification

All radiomic features were used to generate a cluster image map (CIMminer, available at http://discover.nci.nih.gov). The calculations were based on the Euclidian distance method and average linkage cluster algorithm.

### Supervised Classification

For the radiomics analysis, the datasets were standardized by z-score transformation. To test the discriminative power of individual parameters, the Kruskal-Wallis test was performed. The parameters were ranked according to their p-value. The feature stability was tested by performing a Test/Retest and calculating the concordance correlation coefficient, using two images from the same location acquired at different time points. The features were ranked from stable to least stable. Furthermore, the dynamic range of the individual parameters was evaluated. Therefore, the normalized dynamic range was calculated and the parameters were ranked accordingly^35^. The Quade test was performed to analyse the impact of multiple delineations (user independence). The features were ranked from high to low user independence. The four rankings (i.e. discriminative power, feature stability, dynamic range, and user independence) were combined to prepare a final ranking of all features. For each imaging biomarker class the feature with the lowest rank was selected for the radiomic signature. The correlation of these four features was tested by calculating the Pearson correlation coefficient (r-value). If for one or several parameter pairs a correlation coefficient of higher than 0.75 was calculated, only the feature with the higher rank was kept and the other(s) were replaced by a feature from the same imaging biomarker class with the next highest rank. The final four parameters were the radiomic signature that was used for training the linear SVM classification model. A 4-fold cross-validation training scheme was applied for the training of the classification model. The Wilson score interval was calculated to assess the 95% confidence interval of the model.

### Statistical analysis

Statistical analysis was performed using Matlab version 2017b (Mathworks, Natick, MA) and GraphPad Prism 5 (GraphPad Software, La Jolla, CA). The frequency distribution of tumour phenotypes in the different clusters of the unsupervised classification was performed using an exact multinomial test, including a post-hoc test and Bonferroni correction^36^. The data was z-score transformed and analysed with the Kruskal-Wallis test to identify parameters which are significantly different between the three tumour models. The Tukey’s honestly significant difference criterion post-hoc test was performed to depict group differences. The Quade test was performed to calculate the user independence with respect to three different delineations of the tumour. The concordance correlation coefficient was used to determine the feature stability in between to images of the same position. The Pearson correlation coefficient (r-value) was calculated using R^37–39^. A r-value of >0.75 was considered as a high correlation. The Wilson score interval was used to calculate the 95% confidence interval of correct classifications of our supervised learning algorithm.

### Data availability

The datasets generated during and/or analysed during the current study are available from the corresponding author on reasonable request.
